# Molecular Autopsy of Sudden Cardiac Death in the Genomics Era

**DOI:** 10.3390/diagnostics11081378

**Published:** 2021-07-30

**Authors:** Vincenzo Castiglione, Martina Modena, Alberto Aimo, Enrica Chiti, Nicoletta Botto, Simona Vittorini, Benedetta Guidi, Giuseppe Vergaro, Andrea Barison, Andrea Rossi, Claudio Passino, Alberto Giannoni, Marco Di Paolo, Michele Emdin

**Affiliations:** 1 Institute of Life Sciences, Scuola Superiore Sant’Anna, 56124 Pisa, Italy; m.modena@santannapisa.it (M.M.); Alberto.Aimo@santannapisa.it (A.A.); vergaro@ftgm.it (G.V.); passino@ftgm.it (C.P.); alberto.giannoni@santannapisa.it (A.G.); emdin@ftgm.it (M.E.); 2 Fondazione Toscana Gabriele Monasterio, 56124 Pisa, Italy; botto@ftgm.it (N.B.); simona.vittorini@ftgm.it (S.V.); barison@ftgm.it (A.B.); andrea.rossi@ftgm.it (A.R.); 3 Fondazione Pisana per la Scienza Onlus, 56124 Pisa, Italy; 4 Legal Medicine Institute, University of Pisa, 56126 Pisa, Italy; enricachiti@gmail.com (E.C.); marco.dipaolo@unipi.it (M.D.P.); 5 Legal Medicine, USL Toscana Nord Ovest, 55100 Lucca, Italy; benedettaguidi@virgilio.it

**Keywords:** sudden cardiac death, molecular autopsy, genetics, next-generation sequencing

## Abstract

Molecular autopsy is the process of investigating sudden death through genetic analysis. It is particularly useful in cases where traditional autopsy is negative or only shows non-diagnostic features, i.e., in sudden unexplained deaths (SUDs), which are often due to an underlying inherited arrhythmogenic cardiac disease. The final goal of molecular autopsy in SUD cases is to aid medico-legal inquiries and to guide cascade genetic screening of the victim’s relatives. Early attempts of molecular autopsy relied on Sanger sequencing, which, despite being accurate and easy to use, has a low throughput and can only be employed to analyse a small panel of genes. Conversely, the recent adoption of next-generation sequencing (NGS) technologies has allowed exome/genome wide examination, providing an increase in detection of pathogenic variants and the discovery of newer genotype-phenotype associations. NGS has nonetheless brought new challenges to molecular autopsy, especially regarding the clinical interpretation of the large number of variants of unknown significance detected in each individual.

## 1. Introduction

Sudden cardiac death (SCD) refers to the unexpected death of an individual due to an underlying cardiac disease occurring within one hour of onset of symptoms in an apparently healthy subject or, if unwitnessed, in someone known to be in good health up to 24 h before the event [1]. SCD represents 15–20% of all deaths in the general population, with an annual incidence ranging from 40 to 100 per 100,000 person-year [2].

In young subjects, SCD is usually a fatal complication of cardiomyopathies, such as hypertrophic cardiomyopathy (HCM), dilated cardiomyopathy (DCM), and arrhythmogenic cardiomyopathy (ACM), or channelopathies, i.e., disorders affecting ion channels such as long QT syndrome (LQTS), short QT syndrome (SQTS), Brugada syndrome (BrS), and catecholaminergic polymorphic ventricular tachycardia (CPVT). In older individuals, coronary artery disease represents the main cause of SCD, followed by cardiomyopathies, myocarditis, and valve diseases [3].

HCM is characterized by unexplained left ventricular hypertrophy, myocyte disarray, and fibrosis. HCM is due to mutations in genes encoding for sarcomere proteins (like MYBPC3 and MYH7) inherited in an autosomal dominant pattern with incomplete penetrance and variable expressivity [4,5]. DCM-typical features include left ventricular enlargement and fibrotic substitution, leading to systolic dysfunction, conduction system abnormalities, and increased susceptibility to life-threatening arrhythmias. A genetic substrate can be identified in nearly 1/3 of cases, with mutations affecting cytoskeletal proteins being the most commonly found; notably, mutations in LMNA and DES are associated with a particularly arrhythmogenic phenotype [4,5]. ACM is characterized by fibro-fatty replacement of the myocardium involving either one or both ventricles. ACM has an autosomal dominant pattern of inheritance with incomplete penetrance and variable expressivity, with a genetic defect usually affecting cardiac desmosomes (PKP2 and DSP are the genes most frequently involved) [4,5].

Channelopathies are associated with an increased risk of ventricular arrhythmias and SCD in the absence of apparent myocardial structural abnormalities. LQTS is diagnosed in the presence of either a QTc ≥ 480 ms, a LQTS risk score > 3 (a diagnostic score including several items regarding ECG, clinical history and family history), or an unequivocally pathogenic mutation in one of the LQTS-associated genes, such as loss-of-function variants of KCNQ1 (LQTS1) or KCNH2 (LQTS2) or gain-of-function variants of SCN5A (LQTS3) [1,6]. SQTS is characterized either by a QTc ≤ 340 ms or a QTc ≤ 360 ms with at least another suggestive feature among the following: a pathogenic mutation (usually a gain-of-function mutation of potassium channels genes like KCNQ1, KCNH2, KCNJ2), a family history of SQTS or juvenile SCD, or survival from cardiac arrest in the absence of structural heart disease [1,6]. BrS is diagnosed in the presence of a spontaneous or drug-induced (with a sodium channel-blocker) ST-elevation ≥ 2 mm in ≥1 right precordial lead, and generally ensues from a loss-of-function mutation of SCN5A, although a pathogenic variant is found in only 1/3 of cases [1,6]. CPVT is characterized by typical bidirectional or polymorphic ventricular tachycardias, usually triggered by exercise, and most commonly derives from mutations of RYR2 and CASQ2 [1,6]. Besides these channelopathies, there is growing evidence that familial forms of congenital conduction disease (CCD) may predispose to potentially life-threatening arrhythmic events and SCD [4,6,7]. Familial CCD can occur in the context of a structural heart disease, such as LMNA-associated DCM or complex congenital heart diseases (usually due to mutations in genes regulating cardiac development like Nkx2.5, GATA4, TBX5), or in a structurally normal heart (isolated CCD); in the latter case, variants of SCN5A and TRPM4 are the most found [4,6]. Nonetheless, patients with isolated CCD often receive a pacemaker without any further testing; therefore, the real prevalence of genetically determined CCD and the types of genes involved are currently poorly understood. Furthermore, most familial CCD-related genes are usually not included in genetic panels for SUD investigation.

Autopsy aims at identifying the cause of death, which may be particularly relevant for risk prediction in family members. Nonetheless, no definite cause of death is identified by autopsy in up to 40% of SCD [8]. These cases are referred to as sudden unexplained deaths (SUDs) and are mainly related to either microstructural cardiac abnormalities or channelopathies [3]. Microstructural cardiomyopathy changes and cardiac conduction system alterations can be overlooked during a standard forensic examination, despite being possible triggers for life-threatening arrhythmias [7]. Careful histopathological examination of the conduction system by standardized protocols [7] may allow for the identification of several congenital or acquired defects (such as accessory pathways, conduction fibers hypoplasia, dispersion, degeneration, inflammatory infiltration, fibro-fatty substitution), which may be responsible for SUD, especially in infants [7]. These findings are in agreement with data from electrophysiological studies using endo-epicardial mapping in idiopathic ventricular fibrillation survivors, showing conduction abnormalities compatible with microstructural alterations, some of them related to alterations in the Purkinje system, in nearly 2/3 of cases [9]. Post-mortem genetic testing is a complementary tool to a meticulous autopsy aimed at identifying a potential genetic substrate of SUD.

The present review summarizes the scientific evidence on the use of molecular autopsy for SUD investigation. Different than other recently published papers addressing the topic of SCD from a holistic point of view [10,11], in this review we aimed to specifically focus on the use of next-generation sequencing (NGS) technologies for molecular autopsy, reporting both the latest data on NGS application in this context as well as technical aspects regarding data processing and interpretation.

## 2. Methods

We searched PubMed and Scopus in March 2021 with the terms ‘molecular autopsy’ OR ‘next generation sequencing’ OR ‘whole exome sequencing’ OR ‘whole genome sequencing’ AND ‘sudden death’ OR ‘sudden cardiac death’ OR ‘sudden unexplained death’. The present paper is a narrative review, therefore no formal criteria for study selection were adopted.

## 3. Molecular Autopsy

“Molecular autopsy” refers to the processes of post-mortem genetic testing on DNA extracted from blood or tissue collected at autopsy to detect a genetic cause responsible for a SUD. The identification of a potentially pathogenic mutation prompts the screening of surviving relatives, with profound implications for their future clinical management [12]. Moreover, the demonstration of a pathophysiological substrate likely responsible for an otherwise SUD represents an invaluable element for medico-legal inquiries. Nonetheless, a molecular autopsy may be negative or inconclusive even when the most advanced technologies for genetic sequencing are employed. Indeed, not all cases of SUD can be ascribed to genetically determined conditions, and genetic variants of unknown significance are commonly found. Relating the identified gene variant to the phenotype of the deceased and studying the segregation of the variant within the family may be important to establish a definite genotype-phenotype association [13].

## 4. Sanger Sequencing

First-generation sequencing technologies are primarily represented by Sanger sequencing, which uses oligonucleotide primers to search for targeted and previously known DNA regions. Sanger sequencing analysis is performed comparing the patient’s electropherogram against a control one. This approach is easy to employ and has a nearly complete accuracy for the identification of genetic variants [14]. It has been the gold standard for genetic research for almost 3 decades, having been also used for the sequencing of the first human genome (the Human Genome Project), completed in 2003 [15].

Historically, molecular autopsy studies relied on Sanger sequencing to test a few channelopathy-associated genes [10,11]. Despite its simplicity and accuracy, this approach has a high cost per sample and allows for the sequencing of one DNA fragment at a time; hence, it is a low throughput technique (i.e., with low efficiency). Moreover, it is poorly applicable for large-scale genetic screening. Therefore, this approach is inevitably associated with some loss of information about other potential disease-causing genes or gene modifiers, ensuing in an overall low “diagnostic yield”. This term, also known as “mutation detection yield”, indicates the probability that a disease-causing variant is identified and represents a good measure of the efficiency of a genetic test [14] (Table 1).

### Sanger Sequencing in Sudden Cardiac Death

In 1999, Ackerman et al. performed the first molecular autopsy by identifying a novel LQTS pathogenic mutation (KCNQ1) in a 19-year-old who died of anoxic encephalopathy after a near-drowning [16]. Some years later, Chugh et al. tested 5 LQTS-associated genes (KCNQ1, KCNH2, SCN5A, KCNE1, and KCNE2) in 12 SUD cases, and identified the same KCNH2 missense mutation in 2 subjects (17%) [17]. Di Paolo et al. later reported a LQTS-associated mutation in 2 out of 10 cases of juvenile SUD, with a mutation detection yield of 20% [18].

Following studies indicated a lower diagnostic yield: 15% (5 out of 33 SUD cases) in Skinner et al. [19] and 11% (5 out of 44 SUD cases) in Winkel et al. [20], both analysing LQTS genes. Tester et al. conducted the largest molecular autopsy study employing Sanger sequencing so far, including 173 SUD cases tested for the following genes: KCNQ1, KCNH2, SCN5A, KCNE1, KCNE2, RYR2. Mutations in RYR2 were found in 12% of subjects and potentially pathogenic variants in genes associated with LQTS in 15%. Notably, SUD cases with a family history of cardiac events showed a significantly higher mutation prevalence (37% vs. 19%), and the diagnostic yield was even higher (45%) among SUD cases aged < 50 years and with a family history of premature SCD [21].

Overall, these studies showed that a significant proportion of SUD derives from a fatal arrhythmic event caused by a channelopathy, but inter-study mutation detection yield was highly variable, likely reflecting a significant heterogeneity in terms of population examined, DNA source (blood vs. paraffin-embedded tissue), number of genes analysed, and criteria for the attribution of variant pathogenicity.

Nonetheless, the evidence from these studies was deemed sufficient by a consensus document by the Heart Rhythm Society and the European Heart Rhythm Association (HRS/EHRA) on genetic testing for cardiomyopathies and channelopathies to state that comprehensive or targeted (RYR2, KCNQ1, KCNH2, and SCN5A) ion channel genetic testing may be considered in SUD cases to determine the cause of death and facilitate the screening of potentially at-risk relatives, especially when LQTS or CPVT is suspected (class IIb, level of evidence C) [4].

## 5. Next-Generation Sequencing

Massively parallel sequencing technologies, better known as next-generation sequencing (NGS) technologies, have been designed to overtake the barriers of first-generation sequencing [14]. NGS simultaneously analyses millions of small polynucleotide fragments of 50 to 250 base pairs (bp), called “short reads”, allowing for high-throughput sequencing. Sample DNA is cut into fragments of 1000 to 10,000 bp and the NGS reads 50–250 bp from either end of the fragment. Each read is “paired” with the read from the opposite end of the fragment (“paired-end” reads); then, alignment algorithms are employed to line up the series of short reads to the “human reference sequence” (the most widely adopted framework for clinical and research genome sequencing deriving from different ethnic groups) to recreate the whole original DNA sequence. Afterwards, a specific software is used to search for mismatches between the reads and the reference sequence which may underlie a variant of interest, although the attribution of clinical significance requires further investigations. This process is very fast and cost-effective, allowing the entire genome to be sequenced in a matter of few days using just a limited amount of DNA [14,22,23] (Table 1).

NGS technology provides accurate and reliable data for most parts of the genome and has been extensively validated against Sanger sequencing [24]. Guidelines for its use as a diagnostic test in clinical practice are available [25].

There are 3 commercially available NGS platforms (Roche/454, Illumina/Solexa and ABI/SOLiD), with the Illumina/Solexa being the most employed (Supplementary Figure S1). A NGS test can be designed to target a restricted gene panel, the whole exome (WES), the whole genome (WGS), or even RNA sequencing (RNA-seq).

Gene panels can range from tens to thousands of genes and are the preferred test when a specific condition or group of diseases is suspected. Indeed, gene panels are usually selected among genes previously associated with a particular phenotype (“core disease gene list”). This approach aims at maximizing sensitivity, specificity and coverage for the selected genes, hence usually has a higher diagnostic yield than WES or WGS [23]. In case of a less clear phenotype, as in SUD, broader gene panels may be preferable, and WES may show a higher diagnostic yield. The decision on which genes to include in the panel is left to the individual laboratory [23]. SCD studies commonly include genes associated with both channelopathies and cardiomyopathies [26]. The cost of a gene panel is variable depending on customization, but usually lower than WES [23].

WES examines all ~22,000 known protein-coding genes, which constitute 1–2% of the entire genome. WES is employed for genetic testing of phenotypes with a broad differential diagnosis or as a second line test when targeted genetic panels have been inconclusive. The diagnostic yield of WES depends on the tested population and the availability of family members, with a mutation detection yield up to 50% in highly selected cohorts.

WGS covers a great deal of the entire genome, providing information on regulatory, intronic, and intergenic regions. The indications for WGS use are similar to WES. DNA sequencing is more uniform than WES, but the large amount of data provided limits its applicability due to storage and analytical issues. WGS has also a higher cost than WES or gene panels [23].

RNA-seq provides information on targeted RNA transcripts or even the whole transcriptome with an overall accuracy superior to microarrays [27].

### 5.1. Variant Calling, Filtering, Prioritization and Interpretation

**Figure 1:**
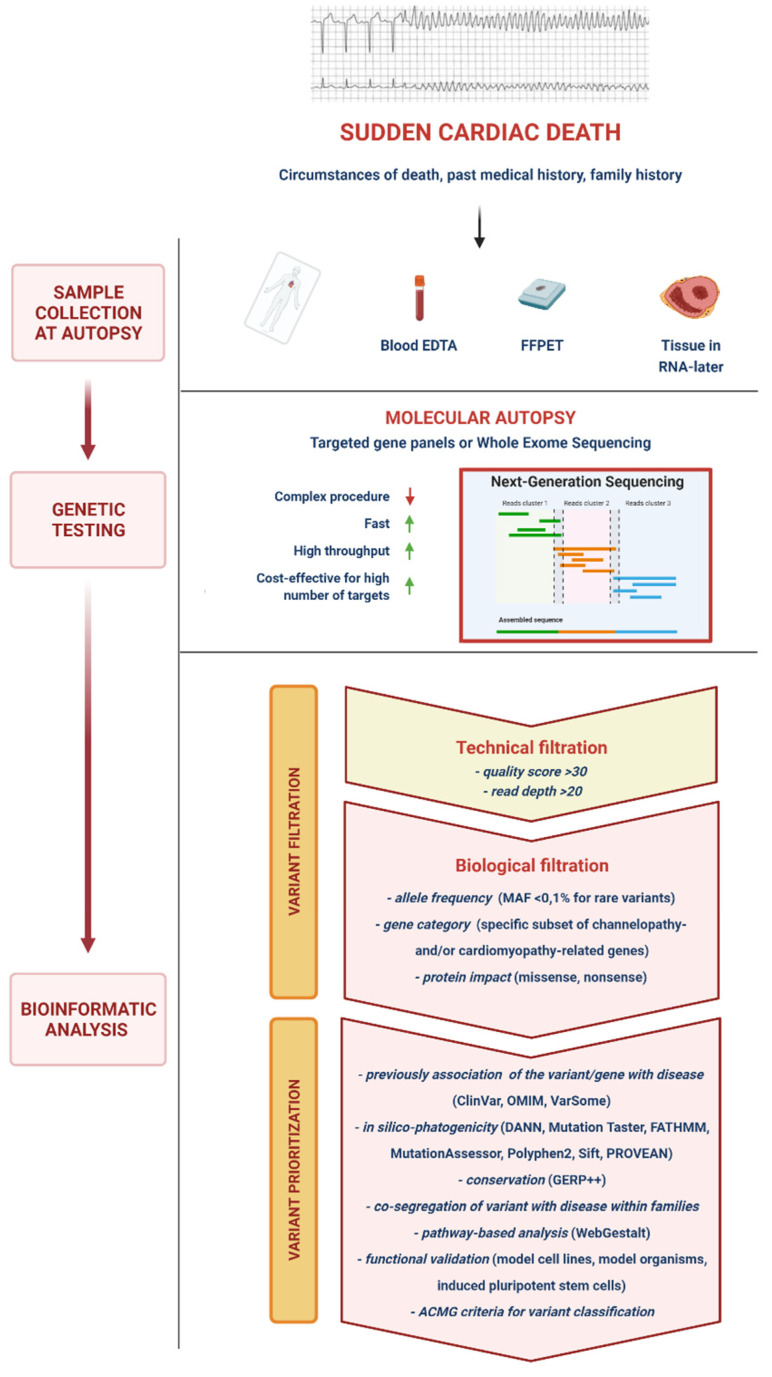
Next-generation sequencing molecular autopsy. DNA of the deceased is extracted from the blood or tissues and then processed via next-generation sequencing tools (usually employing targeted gene pales or exome sequencing), which are faster and more cost-efficient for testing large numbers of genes compared to traditional Sanger sequencing. Once sample DNA sequencing is completed, detected variants must undergo several processes of filtration and prioritization which significantly reduce their number to a small relevant selection for clinical interpretation. ACMG, American College of Medical Genetics and Genomics; DANN, deleterious annotation of genetic variants using neural networks; EDTA, ethylenediaminetetraacetic acid; FATHMM, functional analysis through hidden Markov models; FFPET, formalin-fixed and paraffin-embedded tissue; GERP, genomic evolutionary rate profiling; MAF, minor allele frequency; OMIM, online Mendelian inheritance in man; Polyphen2, polymorphism phenotyping 2; PROVEAN, protein variation effect analyzer; Sift, sorting intolerant from tolerant; WebGestalt, web-based gene set analysis toolkit.

NGS provides a large number of variants which need further filtration and prioritization for clinical interpretation, a process which may differ slightly among individual laboratories, but whose general outline is described below (Figure 1). Several bioinformatic tools are employed in a multistep analysis that produce different files: FASTQ contains base calls of all the reads produced and the quality score of each base; BAM (Binary Aligned/Mapped file) provides read alignment over the reference genome; VCF (Variant Call Format file) includes the chromosomal position, name, and reference genome of each variant.

“Variant calling” is the process of identifying mismatches between the reference genome and the reads aligned over it. There may be errors due to sequencing and alignment mistakes, and specific statistical tools are dedicated to “filter” variants based on the likelihood that a detected mismatch represents a true gene variant or a technical error. Variants are usually identified based on a quality score consisting of a read coverage (i.e., alignment of bases to a specific nucleotide position) ≥ 30-fold and a read percentage (the proportion of bases differing from the reference sequence) ≥ 20. Missense mutations due to single nucleotide polymorphisms are easier to detect through NGS, whereas the probability of finding insertions and deletions of DNA (indels) is inversely proportional to the size of the indel due to higher frequency of alignment errors [22].

After this “technical filtration”, variants must undergo a “biological filtration”. Indeed, rare variants must be differentiated from the large number of missense mutations with no biological relevance present in the general population, described as “background noise”. The ratio of rare variants in the sample DNA to background noise is referred to as “signal-to-noise ratio” [28]. Variants can be filtered for a predefined gene list and/or for a specific frequency (e.g., minor allele frequency —MAF <0.1%, for rare variants) in human genetic databases. In early genomic studies, the absence of a variant in a healthy control population was deemed sufficient to infer its potential pathogenicity, but the novelty of a mutation is no longer considered a reliable criterion for clinical interpretation [28]. Nonetheless, the type of gene involved (e.g., a channelopathy- or cardiomyopathy-associated gene) may provide clues to the clinical relevance of a variant. Another important criterion for biological filtration is the type of mutation, i.e., missense vs. nonsense. Missense mutations are common in unaffected individuals, and genotype–phenotype causal link is more difficult to assess. On the contrary, nonsense mutations (e.g., deletions, insertions and splice-site disrupting mutations) are more likely to produce abnormal proteins and to subsequently have a clinical impact. Accordingly, “nonsense” mutations are rarer and less likely to be found in apparently healthy individuals [28].

After filtration, the variants within a VCF file must be prioritized, i.e., the likelihood that a variant has a functional significance must be established. There are numerous approaches to prioritize variants (Figure 1), and guidelines have been published to standardize this process [29,30]. Previous description of a variant is an important criterion to guide the interpretation of its clinical significance. Databases like ClinVar or OMIM collect information on previously-assessed variants. ClinVar provides a categorical level of evidence for each variant found in scientific literature. Indeed, not all prior reports are robust, and mutations may undergo reclassification as knowledge expands [23]. For example, Campuzano et al. recently reassessed a cohort of 104 subjects and 17 SCD cases diagnosed before 2010 with inherited arrhythmogenic syndromes, finding that more than 70% of rare variants associated with these conditions had changed their classification [31]. In silico tools (DANN, Mutation Taster, FATHMM, MutationAssessor, Polyphen2, Sift, PORVEAN) can be used to predict the effect of a genetic mutation on the protein. Mutations that profoundly alter the protein structure or cause the substitution of an amino acid with another which has completely different chemical properties in a critical domain are more likely to cause a functional change. In particular, aminoacidic substitutions in protein domains conserved in other human proteins with similar function (paralogs) or the same protein in other species (orthologs) are usually more clinically relevant. Dedicated software such as GERP++ or PhyloP can assess intra- and interspecific conservation of DNA sequences [23,28]. Although not always easy to obtain, co-segregation of phenotype with genotype within families is one of the most useful approaches to assess the pathogenicity of a variant.

Most of the previously described approaches are applicable to Mendelian disorders where a single mutation is involved, but there is now evidence that in some cases, particularly in complex phenotypes like SCD, multiple variants may contribute to disease expression [23,28]. Some online tools, like WebGestalt, may be employed to assess if a particular combination of variants may be associated with a particular phenotype. Finally, the functional consequences of mutations can be fully assessed through in vitro cellular expression systems or transgenic animal models. The major drawbacks of these functional studies are their cost and time to obtain results, which make them not suitable for routine evaluation of genetic findings [23,28].

To conclude, the American College of Medical Genetics and Genomics (ACMG) recommends the use of standard terminology to classify variants: ‘pathogenic’, ‘likely pathogenic’, ‘likely benign’, ‘benign’, and ‘variant of unknown significance’ (VUS) [29]. Given the complexity of NGS-related bioinformatic analysis, the continuous advances in the field, and the profound impact that an attribution of pathogenicity of a specific variant can have on the management of an individual, the translation of genetic data into the clinical setting requires specific expertise.

### 5.2. Challenges and Technical Issues

#### 5.2.1. Sample Collection

Blood and fresh frozen tissues are the preferred sources for DNA extraction for genetic analysis. Indeed, the HRS/EHRA consensus paper on genetic testing for channelopathies and cardiomyopathies recommends the collection of “DNA-friendly samples (5–10 mL whole blood in ethylenediaminetetraacetic acid—EDTA—tube, blood spot card, or a frozen sample of heart, liver, or spleen) for subsequent genetic testing” [4]. These samples should be stored refrigerated (<4 weeks) or frozen at −20 °C to −80 °C (>4 weeks) in order to not compromise DNA integrity [32]. Similar recommendations can be found in the recently published Asia Pacific Heart Rhythm Society (APHRS)/HRS consensus paper on the investigation of decedents with SUD and patients with sudden cardiac arrest [33]. Although blood sample storage for future reanalysis is now a common practice in SCD assessment, it is not always available for historical SCD cases, posing limitations to their re-examination. On the contrary, formalin-fixed and paraffin-embedded tissue (FFPET) samples, which are usually prepared for histological analysis, are widely accessible, even for old SCD cases, and may constitute a valuable alternative. However, the process of formalin fixation alters DNA through crosslinking and degradation in fragments of an average length of ~150 bp. Sanger sequencing, which relies on a read length >250 bp, is therefore difficult to perform on FFPET-derived DNA [34]. NGS, thanks to its lower read length, can overcome these limitations. In 2017, Baudhuin et al. were the first to use FFPET samples for genomic evaluation of 4 cases with a clinical phenotype suggestive of an inherited cardiovascular disorder [35]. The same year, Bagnall et al. were the first to demonstrate the feasibility of NGS on FFPET samples from juvenile SCD cases [36]. A recent study compared results of NGS analysis of 12 SCD cases between FFPET and corresponding non-formalin fixed samples (RNA-later-preserved tissues or bloodstain card): all pathogenic variants, likely pathogenic variants or VUS identified in the nonfixed samples were also confirmed in FFPET samples with a variable degree of confidence, but the latter provided more false positives and negatives, particularly when formalin fixation was longer than 8 days [37]. Therefore, caution is advised for the use of FFPET-derived DNA for genomic studies.

#### 5.2.2. Sequencing-Related Issues

NGS does not characterize all areas of the genome with the same precision. Capture approaches for selective sequencing (such as in WES and targeted panels sequencing) and sequencing chemistry itself can lead to uneven DNA coverage, which may cause misinterpretation of variants. For example, areas of the genome rich in cytosine and guanine nucleotides are harder to sequence because the higher-energy bonds between the DNA strands make them less exposed to replication reaction. Since certainty decreases in regions with low coverage, variant calls from these regions are more likely to be discarded [22,23]. NGS is also prone to alignment errors, affecting most commonly areas of insertions or deletions, or regions of the DNA with repeated sequences that are longer than the short reads [22,23]. All these issues are potential sources of false negative results, and underscore that NGS technologies are not perfect, despite continuous improvement in the speed and accuracy of analysis and sequencing.

#### 5.2.3. Variants of Unknown Significance

NGS technologies have significantly increased the number of variants which can be detected in a single individual; therefore, classification of mutations is of the utmost importance to establish a causal link between genotype and phenotype. Despite the fact that several tools are available for variant prioritization, most of them (like large co-segregational studies of functional evaluation of mutations) are not routinely applicable to SUD cases; therefore, with the expansion of gene testing ensues an increase in detection of VUS (mostly missense) [28]. This is currently considered the main drawback of NGS molecular autopsy, since these VUS cannot be used to infer causative relationships and cannot be used for the screening of the victim’s relatives. However, with the more widespread use of NGS and the consequent accumulation of data on SCD-associated variants as well as the development of more sophisticated tools for the prediction of the effect of single or combined mutations, it is expected that numerous VUS will be reclassified in the near future [23,31].

### 5.3. Next-Generation Sequencing in Sudden Cardiac Death

NGS offers the possibility of an “exome molecular autopsy”, changing the perspective from the screening of single genes or small panels to testing large, multigene panels.

In 2014 Bagnall et al. for the first time performed WES in 28 juvenile SUD cases and identified 3 rare variants on major LQTS-associated genes, but the expansion of the panel to other channelopathy- and cardiomyopathy-associated genes led to further identification of 6 rare variants [38]. In a following study, the same group performed gene panel analysis (including either 69, 98 or 101 genes) on 51 SUD cases and WES (filtering for 59 cardiac-related genes) on another 62 SUD cases, finding a mutation in a clinically relevant cardiac gene in 31 cases (27%) [8].

Hata et al. employed a panel of 70 genes to evaluate 25 SUD cases with either normal hearts or non-diagnostic structural abnormalities. They identified 5 known variants and 10 novel variants predicted to have a “high” pathogenic potential after in silico analysis. Mutations involved 3 channelopathy-associated genes (RYR2, CACNA1C, and ANK2), 3 HCM- or DCM-associated genes (MYH7, LDB3, and PRKAG2), 5 ACM-related genes (PKP2, JUP, DSG2, DSP, and TMEM43), and 2 cardiac transcription factor genes (TBX5 and GATA4). Interestingly, combined heterozygous rare variants were found in 3 of the 25 cases, and 2 subjects carried 3 or more variants [39]. These data support the notion that the paradigm of “one gene-one disease” may not apply to all SUD cases, which may sometimes result from the interaction of multiple mutations, as also postulated in a case report by our group [40].

A study on 59 SUD cases included subjects with both autopsy-negative hearts and others with subtle cardiac structural abnormalities not meeting the diagnostic criteria for any specific cardiomyopathy; WES followed by restriction for 135 genes associated with inherited cardiac disorders had a diagnostic yield of 29%, with 7 probands (12%) carrying very rare (MAF < 0.02%) or novel possible pathogenic variants, and 10 (17%) carrying previously published rare (MAF 0.02–0.5%) disease-causing mutations; the higher number of genes tested led to an increase in VUS detection, which were found in 19 (34%) of probands [41]. Hertz et al. employed a panel consisting of 100 channelopathy- and cardiomyopathy-related genes to screen 52 SCD cases with subtle cardiac abnormalities. Variants with “likely functional effects” were identified in 15 cases (29%), of whom 2 (4%) had more than one variant in at least one gene. These mutations were detected with a similar frequency in genes associated with cardiomyopathies (47%) or channelopathies (53%) [42]. These findings confirm the hypothesis that cardiomyopathies may sometimes present with SCD before development of a “diagnostic” phenotype, but also suggest that channelopathies should not be excluded based solely on the presence of minimal structural changes at autopsy.

Ripoll-Vera et al. filtered for a very broad number of genes (194 to 380) related to arrhythmic sudden death for the molecular autopsy of 62 SCDs, obtaining an overall detection yield for pathogenic or probably pathogenic mutations of 31%, at the cost of finding a VUS in about 34% of cases [43]. Dewar et al. published one of the largest studies to date employing a panel of 71 genes in 191 SUDs aged < 5 years. A potentially pathogenic mutation was found in 12 children (6.3%), a novel variant with in silico pathogenic prediction in 15 (7.9%), and a VUS in 36 (18.9%) [44]. Lahrouchi et al. instead employed a panel of 71 genes in 302 SUD cases aged 1–64 years, excluding subjects with evidence of even subtle structural disease. Forty (13%) subjects carried a pathogenic or likely pathogenic mutation, while 42% carried a VUS. Most mutations involved genes associated with LQTS and CPVT, but cardiomyopathy-related genes were also represented. Notably, in surviving relatives the diagnostic yield increased from 26% to 39% thanks to the combination of molecular autopsy and clinical evaluation [26].

Several other post-mortem NGS studies have been recently conducted [45,46,47,48,49,50,51,52,53,54,55,56,57,58], with variable mutation detection rates, not always comparable due to the heterogeneity of genes screened, cases analysed, and methods employed for variant prioritization. Overall, compared with Sanger sequencing-based molecular autopsy, NGS studies have highlighted that cardiomyopathy genes may play a role in some SUD cases, especially in the presence of subtle non-diagnostic cardiac abnormalities, but even in their absence. Moreover, NGS studies have shown the possible coexistence of more than one pathogenic mutation potentially responsible for SCD. Nonetheless, the expansion of genes tested has only slightly improved the overall diagnostic yield of SUD (on average from 20% to nearly 35%), mostly because of the major role still played by the 5 most common channelopathy-related genes (KCNH2, KCNJ2, KCNQ1, RYR2, and SCN5A), which should be tested in all SUD cases. In addition, with the increasing number of genes tested, the majority of additional variants identified tend to be VUS. The reclassification of VUS would be the key to significantly improve the diagnostic yield of NGS molecular autopsy.

## 6. Conclusions

Molecular autopsy is a fundamental aid to forensic examination, aiming at establishing a genetic diagnosis when traditional autopsy is inconclusive, with the final goal of aiding medico-legal investigations and guiding cascade genetic screening of the victim’s relatives. The diagnostic yield of the molecular autopsy is on average 20% with classical Sanger sequencing, but increases up to 35% and more with targeted NGS or WES, at the cost of detecting a larger number of VUS. Despite its undeniable advantages, the relatively low mutation detection yield of molecular autopsy currently prevents it from being a stand-alone tool in the assessment of SUD, which always requires a comprehensive clinical evaluation. Nonetheless, molecular autopsy through genomic technologies offers the possibility to store data for future reassessments which may unveil novel genotype-phenotype associations, thus supporting an extensive use of this approach.

## Figures and Tables

**Table 1:** Comparison of the main features of traditional Sanger sequencing and next-generation sequencing.

Features	Sanger Sequencing	Next-Generation Sequencing
**Approach**	Irreversible chain termination method using dideoxynucleotides (ddNTPs)	Different NGS platform uses different principles (e.g., Illumina—reversible chain termination)
**Throughput**	Low	High
**Sequencing samples**	Clones, PCR	DNA libraries
**Preparation steps**	Few; simple procedure	Many; complex procedure
**Speed**	Slow	Fast (WGS in 2 days)
**Read length**	600–800 bp	35–20,000 bp (depends on the platform)
**Data**	1 read	Millions of reads
**Data accuracy**	99%	99.999%
**Run cost**	Cost-effective for low number of targets	Cost-effective for high number of targets
**Data interpretation**	Electrophoresis gel	Bioinformatic analysis

bp, base pair; ddNTP, dideoxynucleotide; DNA, deoxyribonucleic acid; NGS, next-generation sequencing; PCR, polymerase chain reaction; WGS, whole genome sequencing.

## Supplementary Materials

The following are available online at https://www.mdpi.com/article/10.3390/diagnostics11081378/s1, Figure S1: Illumina/Solexa next-generation sequencing platform.

## References

[1] Priori S.G., Blomstrom-Lundqvist C., Mazzanti A., Bloma N., Borggrefe M., Camm J., Elliott P.M., Fitzsimons D., Hatala R., Hindricks G. (2015). 2015 ESC Guidelines for the Management of Patients with Ventricular Arrhythmias and the Prevention of Sudden Cardiac Death the Task Force for the Management of Patients with Ventricular Arrhythmias and the Prevention of Sudden Cardiac Death of the Europea. Eur. Heart J..

[2] Wong C.X., Brown A., Lau D.H., Chugh S.S., Albert C.M., Kalman J.M., Sanders P. (2019). Epidemiology of Sudden Cardiac Death: Global and Regional Perspectives. Heart Lung Circ..

[3] Eckart R.E., Shry E.A., Burke A.P., McNear J.A., Appel D.A., Castillo-Rojas L.M., Avedissian L., Pearse L.A., Potter R.N., Tremaine L. (2011). Sudden Death in Young Adults: An Autopsy-Based Series of a Population Undergoing Active Surveillance. J. Am. Coll. Cardiol..

[4] Ackerman M.J., Priori S.G., Willems S., Berul C., Brugada R., Calkins H., Camm A.J., Ellinor P.T., Gollob M., Hamilton R. (2011). HRS/EHRA Expert Consensus Statement on the State of Genetic Testing for the Channelopathies and Cardiomyopathies: This Document Was Developed as a Partnership between the Heart Rhythm Society (HRS) and the European Heart Rhythm Association (EHRA). Heart Rhythm.

[5] Buja L.M., Ottaviani G., Mitchell R.N. (2019). Pathobiology of Cardiovascular Diseases: An Update. Cardiovasc. Pathol..

[6] Priori S.G., Wilde A.A., Horie M., Cho Y., Behr E.R., Berul C., Blom N., Brugada J., Chiang C.-E., Huikuri H. (2013). HRS/EHRA/APHRS Expert Consensus Statement on the Diagnosis and Management of Patients with Inherited Primary Arrhythmia Syndromes: Document Endorsed by HRS, EHRA, and APHRS in May 2013 and by ACCF, AHA, PACES, and AEPC in June 2013. Heart Rhythm.

[7] Ottaviani G., Buja L.M. (2016). Anatomopathological Changes of the Cardiac Conduction System in Sudden Cardiac Death, Particularly in Infants: Advances over the Last 25 Years. Cardiovasc. Pathol..

[8] Bagnall R.D., Weintraub R.G., Ingles J., Duflou J., Yeates L., Lam L., Davis A.M., Thompson T., Connell V., Wallace J. (2016). A Prospective Study of Sudden Cardiac Death among Children and Young Adults. N. Engl. J. Med..

[9] Haïssaguerre M., Duchateau J., Dubois R., Hocini M., Cheniti G., Sacher F., Lavergne T., Probst V., Surget E., Vigmond E. (2020). Idiopathic Ventricular Fibrillation: Role of Purkinje System and Microstructural Myocardial Abnormalities. JACC Clin. Electrophysiol..

[10] Orland K.M., Anderson K.B. (2019). Molecular Autopsy for Sudden Cardiac Death: Current State and Considerations. Curr. Genet. Med. Rep..

[11] Bagnall R.D., Singer E.S., Tfelt-Hansen J. (2020). Sudden Cardiac Death in the Young. Heart Lung Circ..

[12] Brohus M., Arsov T., Wallace D.A., Jensen H.H., Nyegaard M., Crotti L., Adamski M., Zhang Y., Field M.A., Athanasopoulos V. (2021). Infanticide vs. Inherited Cardiac Arrhythmias. EP Eur..

[13] Grassi S., Vidal M.C., Campuzano O., Arena V., Alfonsetti A., Rossi S.S., Scarnicci F., Iglesias A., Brugada R., Oliva A. (2021). Sudden Death without a Clear Cause after Comprehensive Investigation: An Example of Forensic Approach to Atypical/Uncertain Findings. Diagnostics.

[14] Goodwin S., McPherson J.D., McCombie W.R. (2016). Coming of Age: Ten Years of next-Generation Sequencing Technologies. Nat. Rev. Genet..

[15] International Human Genome Sequencing Consortium (2004). Finishing the Euchromatic Sequence of the Human Genome. Nature.

[16] Ackerman M.J., Tester D.J., Porter C.J., Edwards W.D. (1999). Molecular Diagnosis of the Inherited Long-QT Syndrome in a Woman Who Died after near-Drowning. N. Engl. J. Med..

[17] Chugh S.S., Senashova O., Watts A., Tran P.T., Zhou Z., Gong Q., Titus J.L., Hayflick S.J. (2004). Postmortem Molecular Screening in Unexplained Sudden Death. J. Am. Coll. Cardiol..

[18] Di Paolo M., Luchini D., Bloise R., Priori S.G. (2004). Postmortem Molecular Analysis in Victims of Sudden Unexplained Death. Am. J. Forensic Med. Pathol..

[19] Skinner J.R., Crawford J., Smith W., Aitken A., Heaven D., Evans C.-A., Hayes I., Neas K.R., Stables S., Koelmeyer T. (2011). Prospective, Population-Based Long QT Molecular Autopsy Study of Postmortem Negative Sudden Death in 1 to 40 Year Olds. Heart Rhythm.

[20] Winkel B.G., Holst A.G., Theilade J., Kristensen I.B., Thomsen J.L., Ottesen G.L., Bundgaard H., Svendsen J.H., Haunsø S., Tfelt-Hansen J. (2011). Nationwide Study of Sudden Cardiac Death in Persons Aged 1-35 Years. Eur. Heart J..

[21] Tester D.J., Medeiros-Domingo A., Will M.L., Haglund C.M., Ackerman M.J. (2012). Cardiac Channel Molecular Autopsy: Insights from 173 Consecutive Cases of Autopsy-Negative Sudden Unexplained Death Referred for Postmortem Genetic Testing. Mayo Clin. Proc..

[22] Priest J.R. (2017). A Primer to Clinical Genome Sequencing. Curr. Opin. Pediatr..

[23] Adams D.R., Eng C.M. (2018). Next-Generation Sequencing to Diagnose Suspected Genetic Disorders. N. Engl. J. Med..

[24] Beck T.F., Mullikin J.C., Biesecker L.G., NISC Comparative Sequencing Program (2016). Systematic Evaluation of Sanger Validation of Next-Generation Sequencing Variants. Clin. Chem..

[25] Bowdin S., Gilbert A., Bedoukian E., Carew C., Adam M.P., Belmont J., Bernhardt B., Biesecker L., Bjornsson H.T., Blitzer M. (2016). Recommendations for the Integration of Genomics into Clinical Practice. Genet. Med. Off. J. Am. Coll. Med. Genet..

[26] Lahrouchi N., Raju H., Lodder E.M., Papatheodorou E., Ware J.S., Papadakis M., Tadros R., Cole D., Skinner J.R., Crawford J. (2017). Utility of Post-Mortem Genetic Testing in Cases of Sudden Arrhythmic Death Syndrome. J. Am. Coll. Cardiol..

[27] Wang Z., Gerstein M., Snyder M. (2009). RNA-Seq: A Revolutionary Tool for Transcriptomics. Nat. Rev. Genet..

[28] Miles C.J., Behr E.R. (2016). The Role of Genetic Testing in Unexplained Sudden Death. Transl. Res. J. Lab. Clin. Med..

[29] Richards S., Aziz N., Bale S., Bick D., Das S., Gastier-Foster J., Grody W.W., Hegde M., Lyon E., Spector E. (2015). Standards and Guidelines for the Interpretation of Sequence Variants: A Joint Consensus Recommendation of the American College of Medical Genetics and Genomics and the Association for Molecular Pathology. Genet. Med. Off. J. Am. Coll. Med. Genet..

[30] Biesecker L.G., Harrison S.M., the ClinGen Sequence Variant Interpretation Working Group (2018). The ACMG/AMP Reputable Source Criteria for the Interpretation of Sequence Variants. Genet. Med. Off. J. Am. Coll. Med. Genet..

[31] Campuzano O., Sarquella-Brugada G., Fernandez-Falgueras A., Coll M., Iglesias A., Ferrer-Costa C., Cesar S., Arbelo E., García-Álvarez A., Jordà P. (2020). Reanalysis and Reclassification of Rare Genetic Variants Associated with Inherited Arrhythmogenic Syndromes. EBioMedicine.

[32] Middleton O., Baxter S., Demo E., Honeywell C., Jentzen J., Miller F., Pinckard J.K., Reichard R.R., Rutberg J., Stacy C. (2013). National Association of Medical Examiners Position Paper: Retaining Postmortem Samples for Genetic Testing. Acad. Forensic Pathol..

[33] Stiles M.K., Wilde A.A.M., Abrams D.J., Ackerman M.J., Albert C.M., Behr E.R., Chugh S.S., Cornel M.C., Gardner K., Ingles J. (2021). 2020 APHRS/HRS Expert Consensus Statement on the Investigation of Decedents with Sudden Unexplained Death and Patients with Sudden Cardiac Arrest, and of Their Families. Heart Rhythm.

[34] Srinivasan M., Sedmak D., Jewell S. (2002). Effect of Fixatives and Tissue Processing on the Content and Integrity of Nucleic Acids. Am. J. Pathol..

[35] Baudhuin L.M., Leduc C., Train L.J., Avula R., Kluge M.L., Kotzer K.E., Lin P.T., Ackerman M.J., Maleszewski J.J. (2017). Technical Advances for the Clinical Genomic Evaluation of Sudden Cardiac Death. Circ. Cardiovasc. Genet..

[36] Bagnall R.D., Ingles J., Yeates L., Berkovic S.F., Semsarian C. (2017). Exome Sequencing–Based Molecular Autopsy of Formalin-Fixed Paraffin-Embedded Tissue after Sudden Death. Genet. Med..

[37] Lin Y., Gryazeva T., Wang D., Zhou B., Um S.Y., Eng L.S., Ruiter K., Rojas L., Williams N., Sampson B.A. (2020). Using Postmortem Formalin Fixed Paraffin-Embedded Tissues for Molecular Testing of Sudden Cardiac Death: A Cautionary Tale of Utility and Limitations. Forensic Sci. Int..

[38] Bagnall R.D., K J.D., Duflou J., Semsarian C. (2014). Exome Analysis–Based Molecular Autopsy in Cases of Sudden Unexplained Death in the Young. Heart Rhythm.

[39] Hata Y., Kinoshita K., Mizumaki K., Yamaguchi Y., Hirono K., Ichida F., Takasaki A., Mori H., Nishida N. (2016). Postmortem Genetic Analysis of Sudden Unexplained Death Syndrome under 50 Years of Age: A next-Generation Sequencing Study. Heart Rhythm.

[40] Modena M., Castiglione V., Aretini P., Mazzanti C.M., Chiti E., Giannoni A., Emdin M., Di Paolo M. (2020). Unveiling a Sudden Unexplained Death Case by Whole Exome Sequencing and Bioinformatic Analysis. Mol. Genet. Genomic Med..

[41] Nunn L.M., Lopes L.R., Syrris P., Murphy C., Plagnol V., Firman E., Dalageorgou C., Zorio E., Domingo D., Murday V. (2016). Diagnostic Yield of Molecular Autopsy in Patients with Sudden Arrhythmic Death Syndrome Using Targeted Exome Sequencing. EP Eur..

[42] Hertz C.L., Christiansen S.L., Ferrero-Miliani L., Dahl M., Weeke P.E., Ottesen G.L., Frank-Hansen R., Bundgaard H., Morling N. (2016). Next-Generation Sequencing of 100 Candidate Genes in Young Victims of Suspected Sudden Cardiac Death with Structural Abnormalities of the Heart. Int. J. Leg. Med..

[43] Ripoll-Vera T., Pérez Luengo C., Borondo Alcázar J.C., García Ruiz A.B., Sánchez Del Valle N., Barceló Martín B., Poncela García J.L., Gutiérrez Buitrago G., Dasi Martínez C., Canós Villena J.C. (2021). Sudden Cardiac Death in Persons Aged 50 Years or Younger: Diagnostic Yield of a Regional Molecular Autopsy Program Using Massive Sequencing. Rev. Esp. Cardiol. Engl. Ed..

[44] Dewar L.J., Alcaide M., Fornika D., D’Amato L., Shafaatalab S., Stevens C.M., Balachandra T., Phillips S.M., Sanatani S., Morin R.D. (2017). Investigating the Genetic Causes of Sudden Unexpected Death in Children Through Targeted Next-Generation Sequencing Analysis. Circ. Cardiovasc. Genet..

[45] Farrugia A., Keyser C., Hollard C., Raul J.S., Muller J., Ludes B. (2015). Targeted next Generation Sequencing Application in Cardiac Channelopathies: Analysis of a Cohort of Autopsy-Negative Sudden Unexplained Deaths. Forensic Sci. Int..

[46] Anderson J.H., Tester D.J., Will M.L., Ackerman M.J. (2016). Whole-Exome Molecular Autopsy After Exertion-Related Sudden Unexplained Death in the Young. Circ. Cardiovasc. Genet..

[47] Rueda M., Wagner J.L., Phillips T.C., Topol S.E., Muse E.D., Lucas J.R., Wagner G.N., Topol E.J., Torkamani A. (2017). Molecular Autopsy for Sudden Death in the Young: Is Data Aggregation the Key?. Front. Cardiovasc. Med..

[48] Buscemi L., Alessandrini F., Perna G., Tagliabracci A. (2015). Next-Generation Sequencing of 68 Genes in Sudden Unexplained Death of Young Individuals in Forensics. Forensic Sci. Int. Genet. Suppl. Ser..

[49] Brion M., Blanco-Verea A., Sobrino B., Santori M., Gil R., Ramos-Luis E., Martinez M., Amigo J., Carracedo A. (2014). Next Generation Sequencing Challenges in the Analysis of Cardiac Sudden Death Due to Arrhythmogenic Disorders. Electrophoresis.

[50] Narula N., Tester D.J., Paulmichl A., Maleszewski J.J., Ackerman M.J. (2015). Post-Mortem Whole Exome Sequencing with Gene-Specific Analysis for Autopsy Negative Sudden Unexplained Death in the Young: A Case Series. Pediatr. Cardiol..

[51] Santori M., Blanco-Verea A., Gil R., Cortis J., Becker K., Schneider P.M., Carracedo A., Brion M. (2015). Broad-Based Molecular Autopsy: A Potential Tool to Investigate the Involvement of Subtle Cardiac Conditions in Sudden Unexpected Death in Infancy and Early Childhood. Arch. Dis. Child..

[52] Christiansen S.L., Hertz C.L., Ferrero-Miliani L., Dahl M., Weeke P.E., LuCamp, Ottesen G.L., Frank-Hansen R., Bundgaard H., Morling N. (2016). Genetic Investigation of 100 Heart Genes in Sudden Unexplained Death Victims in a Forensic Setting. Eur. J. Hum. Genet..

[53] Hellenthal N., Gaertner-Rommel A., Klauke B., Paluszkiewicz L., Stuhr M., Kerner T., Farr M., Püschel K., Milting H. (2017). Molecular Autopsy of Sudden Unexplained Deaths Reveals Genetic Predispositions for Cardiac Diseases among Young Forensic Cases. EP Eur..

[54] Neubauer J., Lecca M.R., Russo G., Bartsch C., Medeiros-Domingo A., Berger W., Haas C. (2018). Exome Analysis in 34 Sudden Unexplained Death (SUD) Victims Mainly Identified Variants in Channelopathy-Associated Genes. Int. J. Leg. Med..

[55] Sanchez O., Campuzano O., Fernández-Falgueras A., Sarquella-Brugada G., Cesar S., Mademont I., Mates J., Pérez-Serra A., Coll M., Pico F. (2016). Natural and Undetermined Sudden Death: Value of Post-Mortem Genetic Investigation. PLoS ONE.

[56] Campuzano O., Beltramo P., Fernandez A., Iglesias A., García L., Allegue C., Sarquella-Brugada G., Coll M., Perez-Serra A., Mademont-Soler I. (2018). Molecular Autopsy in a Cohort of Infants Died Suddenly at Rest. Forensic Sci. Int. Genet..

[57] Shanks G.W., Tester D.J., Ackerman J.P., Simpson M.A., Behr E.R., White S.M., Ackerman M.J. (2018). Importance of Variant Interpretation in Whole-Exome Molecular Autopsy. Circulation.

[58] Mak C.M., Mok N.S., Shum H.C., Siu W.K., Chong Y.K., Lee H.H.C., Fong N.C., Tong S.F., Lee K.W., Ching C.K. (2019). Sudden Arrhythmia Death Syndrome in Young Victims: A Five-Year Retrospective Review and Two-Year Prospective Molecular Autopsy Study by next-Generation Sequencing and Clinical Evaluation of Their First-Degree Relatives. Hong Kong Med. J..

