# Supplementary material for: Molecular Autopsy of Sudden Cardiac Death in the Genomics Era
Source: Diagnostics (Basel). 2021 Jul 30;11(8):1378. doi: 10.3390/diagnostics11081378 (PMC8394514; doi:10.3390/diagnostics11081378)

Supplementary Figure 1. Illumina/Solexa next generation sequencing platform.

Illumina/Solexa is the most employed next generation sequencing (NGS) platform. 1) NGS-library is prepared by DNA fragmentation and tagging with specialized adapters to both fragment ends. 2) The library is loaded into a flow cell and each fragment bound on the flow cell surface is amplified into a clonal cluster. 3) Sequencing reagents are incorporated, and the flow cell is imaged: the emission from each cluster is used to identify the specific base. 4) Through bioinformatics software the reads are aligned to a reference genome and the differences between the reference and the newly sequenced reads can be identified.

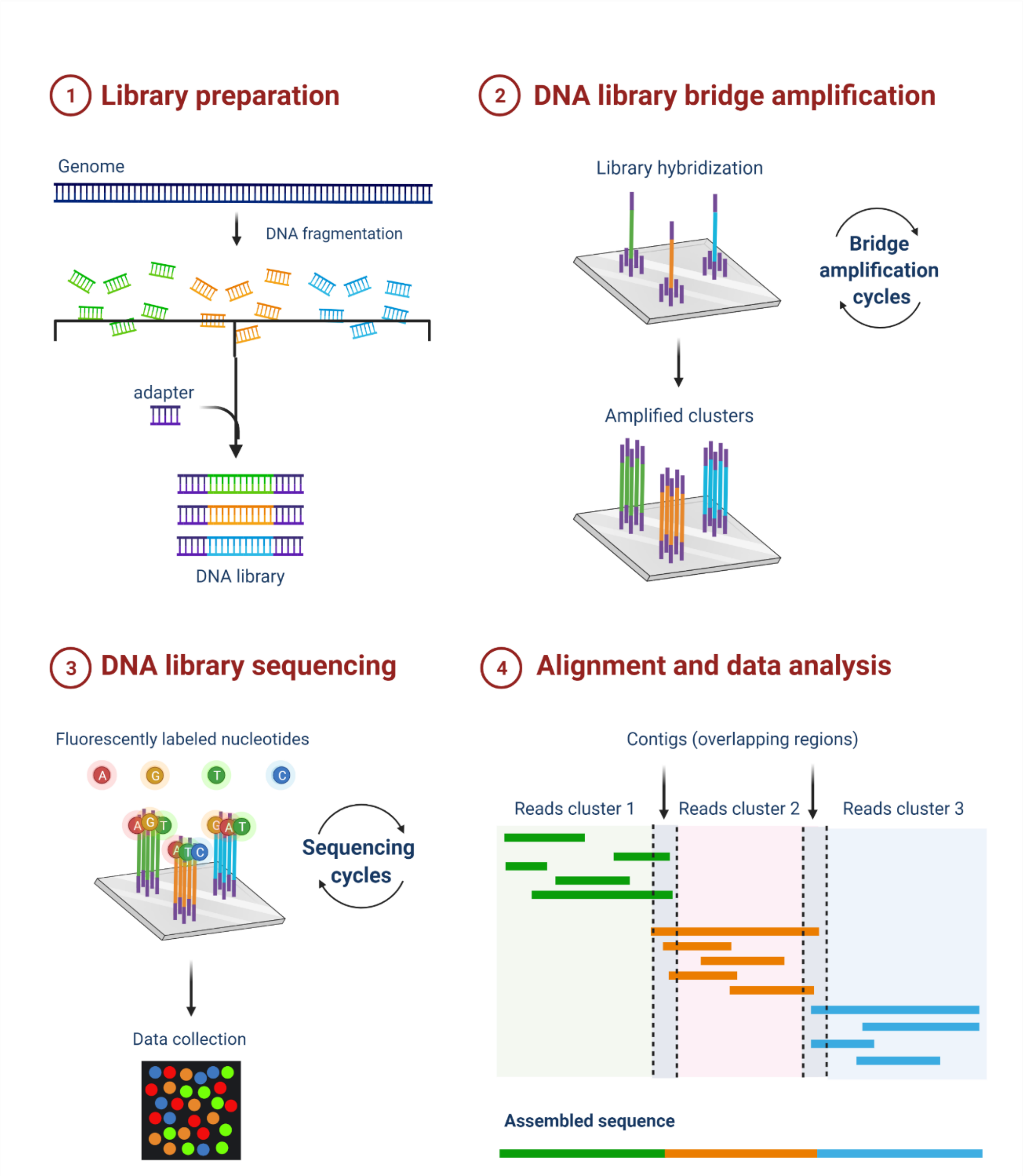

Supplement: Supplementary file 1 [file diagnostics-11-01378-s001.zip › diagnostics-1248769-Supplementary.pdf]
